# Modulation of Glucose Takeup by Glucose Transport on the Isolated OHCs

**DOI:** 10.1155/2018/7513217

**Published:** 2018-04-05

**Authors:** Xiao-ting Cheng, Feng-bo Yang, Qing-qing Jiang, Rong Zhang, Shi-ming Yang, Ning Yu

**Affiliations:** ^1^Department of Otorhinolaryngology Head and Neck Surgery and Institute of Otorhinolaryngology, The PLA General Hospital, Beijing 100853, China; ^2^Department of Otorhinolaryngology Head and Neck Surgery, The First Affiliated Hospital of Fujian Medical University, Fujian 350005, China; ^3^Department of Otolaryngology, Affiliated Hospital of North Sichuan Medical College, Nanchong 637100, China

## Abstract

Glucose is a fundamental source of energy for mammalian cells; however, whether glucose is taken up through the lateral walls of cochlear outer hair cells (OHCs) is unknown. The OHC lateral wall is complex, composed of a plasma membrane, cortical lattice, and subsurface cisternae. This study assessed the uptake of glucose by OHCs using live-cell microscopy and examined the distribution of glucose transporter isoforms by immunohistochemistry. We found that glucose transporter-4 was mostly expressed on the lateral wall of OHCs. Glucose crossed the lateral walls of OHCs via glucose transporters-4 mainly, and this process could be modulated. These results suggest that the lateral walls are involved in modulating energy transport into OHCs.

## 1. Introduction

The length of cochlear OHCs can change at a very high frequency when they receive acoustic signals [1, 2]. Although electromotility is not directly dependent on ATP or other chemical intermediates [3, 4], these rapid changes in body length must consume a great deal of energy. The complex lateral cortex of an OHC is composed of three distinct layers, namely, the subsurface cisternae (SSC), cortical cytoskeletal lattice (CL), and plasma membrane (PM) [5]. The SSC is a specialized and substantial fraction of the endoplasmic membrane within OHCs; in guinea pigs, as many as twelve ordered layers line the lateral cytoplasmic surface of the PM [6]. The cortical lattice is an unusual protein skeleton associated with the outermost surface of the lateral cisternae beneath the PM. The outermost PM contains a high density of integral membrane proteins. One of these, prestin, a specialized electromotor protein, is expressed on the outer PM [7]. The relationship between glucose transporters and prestin is unclear [8, 9].

The functions of OHC lateral walls are understudied. How glucose is transported across the three membrane structures, whether they participate in energy transport, and whether glucose transporters are related to electromotility are unknown. Here, we assessed the cytoplasmic uptake of glucose by OHCs using live-cell microscopy and examined the subcellular localization of glucose transporter (GLUT) isoforms by immunohistochemistry. We observed that glucose crossed the membrane via glucose transporters and that this process could be controlled; further, we found that GLU-4 was expressed on the subcellular localization. These results imply that the lateral walls of cochlear OHCs are involved in controlling energy transport.

## 2. Materials and Methods

### 2.1. Animal Preparation and Cochlear Cell Isolation

Cochlear cells were freshly isolated from adult guinea pigs (250–300 g) [10, 11]. Briefly, guinea pigs were anesthetized with an overdose of chloral hydrate (0.3 ml/100 g), and otic capsules were removed after decapitation. The otic capsules were dissected, and the isolated cochleas were put in normal extracellular solution (142 mM NaCl, 5.37 mM KCl, 1.47 mM MgCl_2_, 2 mM CaCl_2_·2H_2_O, and 10 mM HEPES; 300 mOsm; pH 7.2). After removal of the bone and stria vascularis, the sensory epithelium (organ of Corti) was collected using a sharpened needle and further dissociated with trypsin (0.5 mg/ml) for 2-3 min with shaking. Cochlear cells were then transferred to a recording dish. All experimental procedures were performed at room temperature (23°C). The animals were purchased from Vital River Laboratory Animal Technology Co. Ltd. (Beijing, China). All animal experiments were approved by the Institutional Animal Care and Use Committee of the Chinese PLA General Hospital (Beijing, China).

### 2.2. Experimental Agents

Adenosine 5′-triphosphate (ATP) disodium salt solution (cat. no. A6559-25UMO), indinavir (cat. no. Y-0000788), and WZB-117 (SML0621-5MG) were purchased from Sigma (St. Louis, MO). Antibodies against 4-[[[[4-(1,1-dimethylethyl)phenyl]sulfonyl]amino]methyl]-N-3-pyridinylbenzamide (STF-31; cat. no. SC-364692) and prestin (H-294; cat. no. sc-30163) were purchased from Santa Cruz Biotechnology (Santa Cruz, CA). The dyes di-8-ANEPPS (D3167), Hoechst 33342 (R37605), and 2-NBDG (N13195) were purchased from Molecular Probes (Eugene, OR). Antibodies against GLUT-1 (ab652) and GLUT-4 (ab654) were purchased from LifeSpan BioSciences Inc. (Seattle, WA). A perfusion system was used to apply reagents to cells. The animals were anesthetized for all procedures with an intraperitoneal injection of chloral hydrate.

### 2.3. Immunohistochemistry

Dissociated cochlear cells were fixed with 4% paraformaldehyde for 30 min. After three washes with 0.01 M PBS, the cells were incubated in blocking solution (10% goat serum in PBS with 0.25% Triton X-100) for 30 min. The cells were then incubated with anti-GLUT or anti-prestin antibodies (1 : 100–250) in blocking solution at room temperature (23°C) for 2 h. In control experiments, the primary antibody was omitted. After three washes with PBS, the cells were incubated with Alexa Fluor 488-conjugated donkey anti-rabbit IgG (1 : 200; cat. no. A21206, Molecular Probes) or Alexa Fluor 568-conjugated donkey anti-goat IgG (1 : 200; cat. no. A11057, Molecular Probes) in blocking solution at room temperature for 1 h. For costaining with di-8-ANEPPS to visualize the PM and cytoplasmic membranous organelles, the cells were further incubated in 30 *μ*M di-8-ANEPPS (D-3167; Molecular Probes) for 20 min after secondary antibody incubation. After completely washing out the dye with 0.01 M PBS, staining was observed under a confocal microscope.

Cell nuclei were stained with Hoechst 33342 (R37605; Molecular Probes). Following incubation with the secondary antibody, pieces of dissociated cells were incubated with a dilution of Hoechst 33342 stock solution (2 drops per ml) at room temperature for 15–30 min and washed three times with PBS.

### 2.4. Live-Cell Imaging, Image Processing, and Statistical Analysis

Live-cell imaging was performed using an Applied Precision DeltaVision microscope (GE Healthcare, Issaquah, WA), and data were acquired and processed with the accompanying software. Fluorescence intensity was measured using ImageJ. All data are expressed as the mean ± standard deviation (SD). Data were analyzed using an unpaired *t*-test (Student's *t*-test) for two groups and a one-way ANOVA with Bonferroni post hoc tests for multiple comparisons, as noted in the figure legends (SPSS 13.0 software; SPSS Inc., Chicago, IL). Differences with *p* < 0.05 were deemed statistically significant. Error bars in the figures represent the SD.

## 3. Results

### 3.1. 2-NBDG Is Transported into the Cytoplasm of OHCs

**Figure 1:**
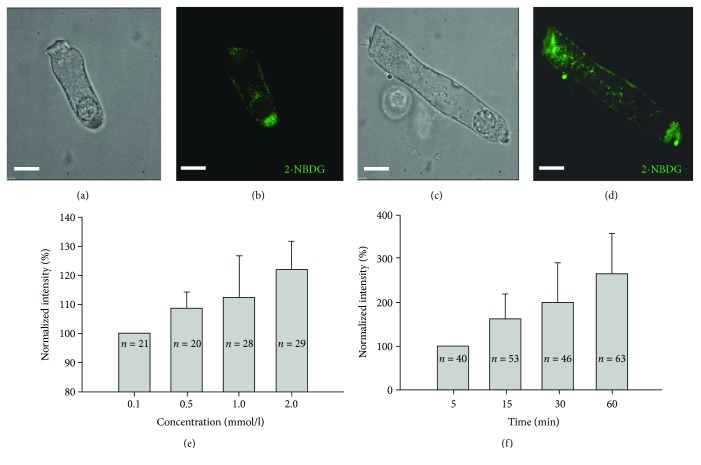
Uptake of 2-NBDG into OHCs and concentration or time-dependent uptake of 2-NBDG by OHCs. (a and c) Nomarski images. (b and d) Guinea pig OHCs of various lengths following exposure to 2-NBDG (1 mM for 20 min; cells were imaged after a 10 min washout; green, 2-NBDG). (e) Normalized fluorescence intensity of 2-NBDG at varying concentrations. The concentration was measured at 20 min after giving the 2-NBDG. (f) Normalized fluorescence intensity of 2-NBDG over time. The concentration of 2-NBDG used was 1 mM/l. Scale bars, 10 *μ*m.

2-NBDG is a novel fluorescent derivative of glucose that is useful for assessing glucose uptake activity [12]. A good viability of isolated OHCs can be sustained for two hours in normal extracellular solution [13], and the procedure for acquiring isolated OHCs in this experiment only need 15 minutes. As shown in Figure 1, following 20 min of exposure to 2-NBDG (1 mM), the OHCs from the basal and apical turn displayed intracellular fluorescence, indicating uptake (Figures 1(b) and 1(d)). Control OHCs without perfusion with 2-NBDG had minimal cellular fluorescence, indicating relatively little autofluorescence (data was not shown here). Hence, the OHCs appeared to take up 2-NBDG rapidly and retain it on the lateral wall and within the cytoplasm (Figures 1(b) and 1(d)).

Then we examined the concentration and time dependency of 2-NBDG uptake by OHCs. We perfused a series of 2-NBDG concentrations (0.1 mm/l, 0.5 mm/l, 1 mm/l, and 2 mm/l) to the OHCs that were isolated from all turns of the cochlea. The normalized fluorescence intensity (NFI) on the lateral wall and cytoplasm at each concentration was divided by the initial concentration (0.1 mm/l) (Figure 1(e)). The NFI increased with the increase of 2-NBDG concentration. Similarly, fluorescence was normalized by dividing the value by that at time zero. The uptake of 2-NBDG increased gradually over time until reaching a plateau (Figure 1(f)). The results suggested that 2-NBDG uptake by OHCs depended on the concentration and time.

### 3.2. GLUT-1 and GLUT-4 Expression in OHCs

**Figure 2:**
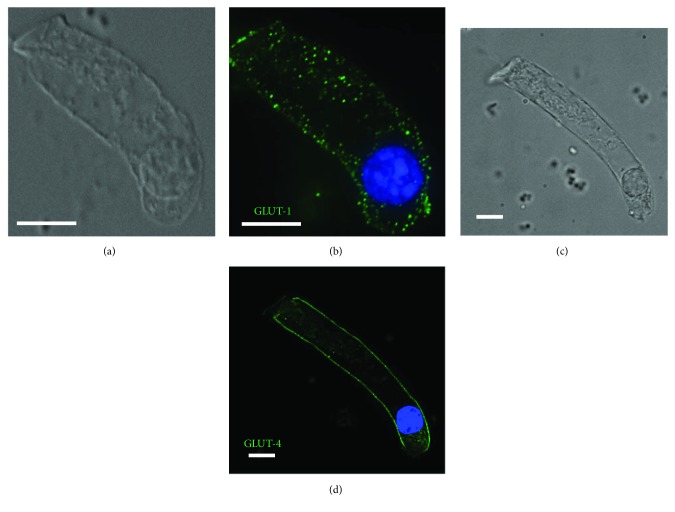
Subcellular distribution of GLUT isoforms in OHCs. Deconvoluted images of OHCs labeled with antibodies specific for various glucose transporters (green). (b) GLUT-1 and (d) GLUT-4. Blue staining corresponds to Hoechst 33342 (nuclei). (a) and (c) are Nomarski images. Scale bar, 10 *μ*m.

Despite the above results showing that 2-NBDG is taken up by OHCs, little is known about the expression and localization of glucose transporters in these cells. Previous studies have shown GLUT-1, GLUT-3, GLUT-4, GLUT-5, GLUT-8, GLUT-10, and GLUT-12 expressions in both the stria vascularis and the spiral ligament [14]. We examined the relative distribution of GLUT isoforms in OHCs by immunohistochemistry (Figure 2; some data was not shown). Staining for all glucose transporters except GLUT-4 was punctate and mostly close to the edge of the cell; GLUT-4 was distributed evenly along the lateral cell edges, similar to previous observations of prestin distribution [7]. We also observed the expression of transporters on the cuticular plate, which has not been previously reported and is consistent with our observation of 2-NBDG uptake by OHCs through the cuticular plate using live-cell imaging (data not shown). While previous reports indicated that OHCs did not express GLUT-5 [9], we observed that the outer hair cells expressed GLUT-5 scatteredly (data was not shown). These findings strongly suggest that a number of GLUT isoforms participate in glucose transport in OHCs.

### 3.3. OHC Uptake of 2-NBDG Is Modulated by GLUT-1 and GLUT-4 Antagonists

**Figure 3:**
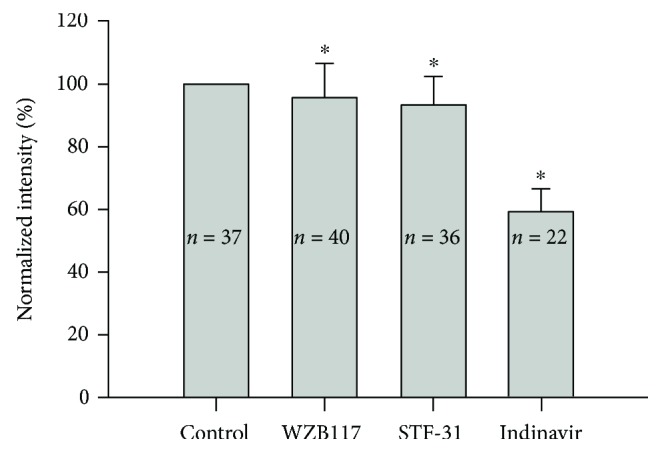
The antagonists of GLUT-1 and GLUT-4 alter the uptake of 2-NBDG. WZB117 and STF-31 are GLUT-1 antagonists and indinavir is a GLUT-4 antagonist. Error bars represent the standard error. ∗ indicates *p* < 0.05 by a paired *t*-test.

We next examined the effect of glucose antagonists on the uptake of 2-NBDG (Figure 3; all data are relative to untreated cells). STF-31 and WZB117 are GLUT-1 antagonists [15, 16] and decreased the uptake of 2-NBDG to 0.9308 ± 0.0909 and 0.9561 ± 0.1095, respectively, though the reductions were small. Indinavir (50 *μ*mol/l) [17, 18] is a GLUT-4 antagonist and decreased the uptake of 2-NBDG to 0.5907 ± 0.0649.

### 3.4. Comparison Dye or Antibody Staining Localization between GLUT-4 and Di-8-ANEPPS

**Figure 4:**
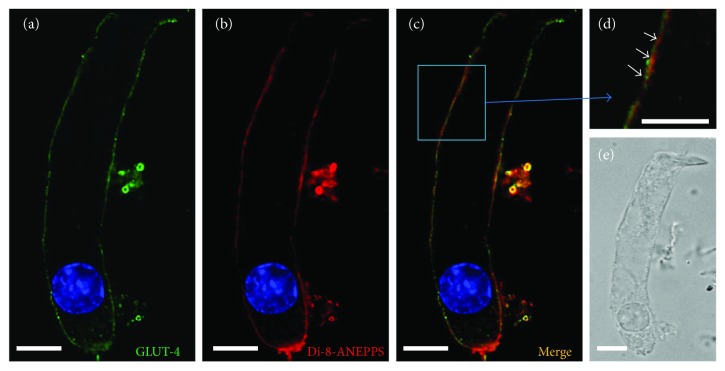
Basal staining for GLUT-4 may result from intracellular penetration of the antibody. OHCs were costained using anti-GLUT-4 antibodies and di-8-ANEPPS: (a) GLUT-4 staining; (b) di-8-ANEPPS staining; (c) merged image; (d) high magnification; (e) Nomarski image. Scale bar, 10 *μ*m in all images except the inset (2 *μ*m).

**Figure 5:**
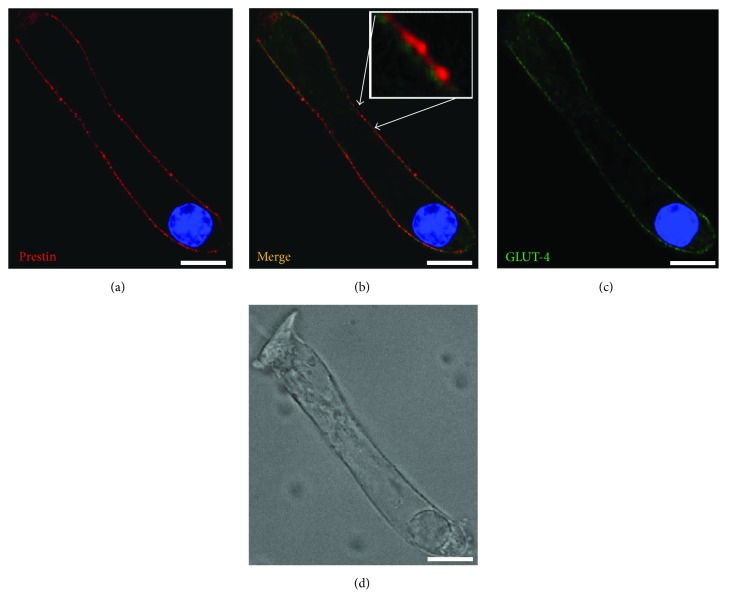
GLUT-4 colocalizes with prestin in the lateral, but not the basal, wall of OHCs. OHCs were costained for GLUT-4 and prestin. (a) Prestin and (b) merged image. Inset: high-magnification image revealing the separation of GLUT-4 and prestin. (c) GLUT-4 and (d) Nomarski image in the same field. Scale bar, 10 *μ*m in all images except the inset (2 *μ*m).

In addition to the lateral walls, some GLUT-4 labeling was observed at the basal PM, though the staining was weak (Figures 2(d), 4(a), and 5(c)). To determine whether the basal staining resulted from GLUT-4 antibody penetration through the PM into the cytoplasm, we costained cells with di-8-ANEPPS, a membrane-impermeable dye that labels the phospholipid bilayer (Figure 4(b)).

Di-8-ANEPPS labeled the PM as well as other cell membranous structures, including the SSC, and overlapped with GLUT-4 labeling at the basolateral wall (Figures 4(a)–4(c)). To clarify the localization of GLUT-4, we colabelled cells using anti-prestin antibodies. Prestin was expressed only in OHCs at the outermost layer of the PM along the lateral wall. High-magnification images (inset in Figure 5(b)) revealed that the two proteins did not colocalize; prestin was confined to the outermost PM layer, while GLUT-4 was just inside, likely at the SSC.

**Figure 6:**
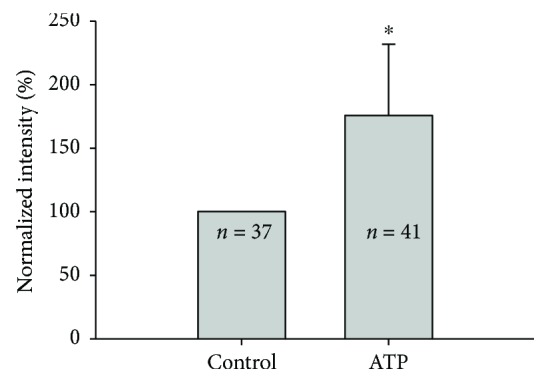
P2X7 receptor agonist alters the uptake of 2-NBDG. ATP is a P2X7 receptor agonist that could increase the uptake of 2-NBDG obviously. Error bars represent the standard error. ∗ indicates *p* < 0.05 by a paired *t*-test.

We next examined the effect of receptor P2X7 agonists on the uptake of 2-NBDG (Figure 6; all data are relative to untreated cells). Intracochlear ATP is an important mediator in the regulation of hearing, and it affects the transmembrane potential in OHCs [19, 20]. The ATP receptor P2X7 is expressed on the lateral wall of OHCs [10, 11], similar to the distribution of GLUT-4 (data below). Thus, we examined the effect of ATP on 2-NBDG uptake by OHCs. ATP increased the uptake of 2-NBDG significantly to 1.7582 ± 0.5625.

## 4. Discussion

In this study, we demonstrated the uptake of 2-NBDG by OHCs. We further demonstrated that the uptake was time- and concentration-dependent and regulated by ATP receptors and glucose transporters.

Our examination of the distribution of GLUT isoforms using specific antibodies identified the expression of glucose transporters on the cuticular plate, in accordance with our live-cell imaging results showing glucose uptake at this location (data not shown). GLUT-1 expressed on all parts of OHCs scatteredly; it was not only in the cytoplasma but also on the cell membranes. The GLUT-1 inhibitors STF-31 and WZB117 [15, 21] inhibited the uptake of 2-NBDG, indicating that glucose are transported through GLUT-1. There is no study showing the GLUT expression in the OHCs; only some studies elucidate GLUT expression in the stria vascularis and the spiral ligament. Takeuchi and Ando [22] and Ando et al. [23] showed that GLUT-1 was expressed in the stria vascularis, whereas in basal cells and not marginal cells, the studies indicated that GLUT-1 might contribute to the transcellular glucose takeup pathway. Edamatsu et al. [14] showed that GLUT-1, GLUT-3, GLUT-5, GLUT-8, GLUT-10, and GLUT-12 were detected in both the stria vascularis and the spiral ligament and revealed that there were significant differences between the stria vascularis and the spiral ligament of GLUT-1, GLUT-4, GLUT-5, and GLUT-10 isoforms, which indicated GLUT-1, GLUT-4, GLUT-5, and GLUT-10 isoforms involved in glucose transport in the stria vascularis and the spiral ligament.

In this study, we observed that the outer hair cells expressed GLUT-5 scatteredly (data was not shown), which was consistent to the results of Belyantseva et al. [24]. They found that GLUT-5 and prestin were highly expressed in the lateral membrane of OHCs, indicating that GLUT-5 might be involved in the control of cochlear electromotility, whereas the results were different from Wu et al. [9] which showed that GLUT-5 was not detected in the OHCs and GLUT-5 was not necessary for OHC motility or cochlear amplification. The discrepancy may due to the different types of experimental animals. Our observation of GLUT-4 expression along the entire lateral wall of OHCs suggests that it is responsible for most glucose transport in these cells. Then we used the GLUT-4 antagonist indinavir to exam the function of GLUT-4 and we observed that the uptake of 2-NBDG decreased when the indinavir was applied. Through these results, we can suggest that GLUT-4 mainly relates with glucose transport in OHCs.

At the same time, we observed that GLUT-4 was localized to a layer just inside the membrane where prestin was expressed, namely, the inner SSC of the OHC lateral wall [7]. Ning et al. [25] showed that the distribution of the P2X7 receptor overlapped with the prestin expression. Thus, the subcellular distribution of GLUT-4 in OHCs also resembled that of P2X7. Prestin is a unique ATP- and Ca^2+^-independent motor protein of the outer hair cell, involved in the electromotility and cochlear amplification [26–28]. The process needs a lot of energy consumption provided by ATP, which is also the P2X7 receptor agonist [29, 30]. This study showed that the uptake of 2-NBDG increased after the application of ATP. ATP enhanced glucose uptake, suggesting that GLUT-4 and P2X7 may participate in electromotility. The distribution of GLUT-4 also suggests that it is related to electromotility; however, more evidence is needed to verify this hypothesis.

Diabetes is a known risk reason for hearing loss, and some previous studies suggest that GLUT-4 mutations are linked to diabetes. GLUT-4 plays an important role in the pathophysiology of T2DM, and its defective expression will hinder the entrance of glucose into the cell for energy production [31–33]. Altered insulin sensitivity may influence the expression of GLUT-4, leading to the death of OHCs because of glucose deficiency. Our observation that GLUT-4 was the most strongly expressed GLUT isoform in OHCs may explain why diabetes can cause deafness. Degerman et al. [34] found that the sensory epithelium of human saccule expressed insulin-sensitive glucose transporter (GLUT-4), which also exhibited expression of the insulin receptor, insulin receptor substrate 1, and protein kinase B, indicating that those proteins might have a role in mechanism between diabetes and hearing loss. Thus, GLUT-4 may participate in the mechanism of hearing impairment of diabetes.

In summary, glucose is transported into OHCs across the lateral membrane via glucose transporters 1 and 4 mainly, and this transport is regulated by ATP and glucose antagonists. GLUT-4 is expressed on the inner OHC lateral walls, suggesting that it is related to electromotility. In conclusion, the lateral walls of cochlear OHCs are involved in controlling energy transport.
